# Endogenous toxic metabolites and implications in cancer therapy

**DOI:** 10.1038/s41388-020-01395-9

**Published:** 2020-07-24

**Authors:** Namgyu Lee, Meghan E. Spears, Anne E. Carlisle, Dohoon Kim

**Affiliations:** Department of Molecular, Cell and Cancer Biology, University of Massachusetts Medical School, Worcester, MA 01605 USA

**Keywords:** Cancer metabolism, Cancer therapy

## Abstract

It is well recognized that many metabolic enzymes play essential roles in cancer cells in producing building blocks such as nucleotides, which are required in greater amounts due to their increased proliferation. On the other hand, the significance of enzymes in preventing the accumulation of their substrates is less recognized. Here, we outline the evidence and underlying mechanisms for how many metabolites normally produced in cells are highly toxic, such as metabolites containing reactive groups (e.g., methylglyoxal, 4-hydroxynonenal, and glutaconyl-CoA), or metabolites that act as competitive analogs against other metabolites (e.g., deoxyuridine triphosphate and l-2-hydroxyglutarate). Thus, if a metabolic pathway contains a toxic intermediate, then we may be able to induce accumulation and poison a cancer cell by targeting the downstream enzyme. Furthermore, this poisoning may be cancer cell selective if this pathway is overactive in a cancer cell relative to a nontransformed cell. We describe this concept as illustrated in selenocysteine metabolism and other pathways and discuss future directions in exploiting toxic metabolites to kill cancer cells.

## Introduction

Metabolism is an aspect of cancer biology that is attractive in terms of therapy. First, it has been known for a long time that the metabolism of cancer cells differs from that of normal cells in many ways. A widely known metabolic alteration in cancer cells is high glucose consumption and high levels of lactate production with a lack of oxidative phosphorylation, referred to as the Warburg effect [1, 2]. Another commonly observed metabolic perturbation in cancer cells is the deregulated uptake of amino acids [3]. In particular, many cancer cells are highly dependent on glutamine for their survival and proliferation [3]. In addition, lipid metabolism is also modified in cancer cells [4] because rapidly proliferating cells require fatty acids for the synthesis of signaling molecules and membranes [5]. The identification of such cancer-specific metabolic changes provides the opportunity to develop novel therapeutic strategies to treat cancer. The “druggability” of enzymes further adds to the appeal of cancer metabolism as a therapeutic avenue. Even if cancer-selective targets are identified by characterizing the role of the targets in cancer, it can be difficult to translate the basic research into the clinic if the targets are not easily druggable, i.e., have small, hydrophobic pockets in regions required for their activity. Enzymes are, by their catalytic nature, highly druggable, due to their pockets for their substrates and coenzymes [6, 7].

**Fig. 1:**
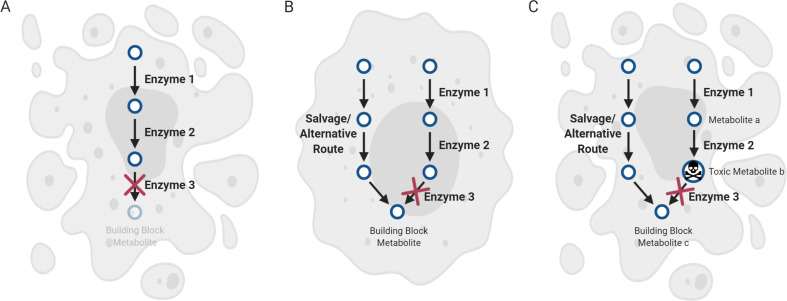
Scenarios for targeting metabolic enzymes that produce essential cellular building blocks in cancer. **a** Targeting a metabolic enzyme to disrupt the production of a metabolite that is essential to a cancer cell can be an effective therapeutic strategy. **b** When there are alternate means for production or acquisition of an essential metabolite, targeting the synthesizing enzyme may be inadequate to kill a cancer cell. **c** An alternative approach is to target an enzyme directly downstream of a toxic metabolite, which will result in accumulation of the upstream toxic metabolite. Even if there are alternative routes for producing the building block metabolite, this strategy should still work to exert toxicity in a cancer cell.

Much of cancer metabolism research has centered on the idea of targeting the “cellular building blocks” that cancer cells require, and there are notable examples of clinical efficacy (Fig. 1a). Cancer cells upregulate a variety of metabolic pathways involved in the production of cellular building blocks that support the increased demand for the biosynthesis of proteins, lipids, and nucleic acids [8]. Antifolates, folate analogs that inhibit de novo nucleotide synthesis enzymes [9, 10], were among the very first chemotherapeutics. Since then, many additional therapies that inhibit nucleotide synthesis have been developed and are still used in the clinic to treat several cancers [11]. Two important examples are the use of 5-fluorouracil, which disrupts thymidine synthesis through the enzyme thymidylate synthase and gemcitabine, which can incorporate into DNA and targets deoxyribonucleotide synthesis through the enzyme ribonucleotide reductase, both of which are required for essential DNA synthesis in rapidly growing cancer cells [11]. In addition to targeting nucleotide synthesis, other biosynthetic pathways have been actively explored and have shown promise in preclinical models, such as PHGDH required for serine biosynthesis and FASN required for fatty acid biosynthesis [12–14].

While the approach of starving a cancer cell of essential metabolites is both logical and proven, there are also some important factors that can limit the effectiveness of this strategy in killing a cancer cell. First, when the biosynthetic pathway for an essential metabolite is disrupted, there may be mechanisms by which a cell can salvage it through a secondary route (Fig. 1b). This can occur by compensatory production of the metabolite through an alternative pathway, uptake from the extracellular environment, or breakdown of existing macromolecules in the cell [8]. Such salvage pathways exist for many key metabolic pathways in the cell, such as nucleotide and sphingolipid biosynthesis. For both of these pathways, in addition to the de novo biosynthesis of essential intermediates like purines/pyrimidines and ceramides, respectively, cells can also obtain these metabolites through extracellular uptake from what is obtained in the diet, as well as degradation of more complex molecules in the cell such as nucleic acids and sphingomyelin [11, 15]. Cancer cells can also upregulate processes such as macropinocytosis [16] and autophagy [8], further contributing to their metabolic flexibility in obtaining building blocks. Therefore, the potential for multiple sources of essential building blocks can make targeting a biosynthetic enzyme insufficient to limit a metabolite adequately to kill a cancer cell.

Second, a metabolic enzyme that appears to be essential in tissue culture may not end up being essential in a more physiological (in vivo) context, as typical culture media conditions do not accurately reflect the diversity and quantity of metabolites that are available in the human bloodstream [17]. Uric acid, pyruvate, taurine and various other metabolites are present at higher levels in human serum than in culture media [17]. Consequently, disrupting a biosynthetic pathway may sufficiently deprive cells of an essential metabolite in tissue culture, while in vivo the cells may be able to obtain the metabolite from the bloodstream.

As illustrated, there are multiple reasons why targeting a particular enzyme that produces cellular building blocks may not be effective to kill a cancer cell. Thus, even when this enzyme appears to be important to a cancer cell, either due to the product it makes or due to it being increased in expression and/or activity, targeting it may not translate to effective therapy. Here, we outline an alternative approach: the notion of killing cancer cells not by impairing the production of biosynthetic building blocks, but rather by inducing an accumulation of toxic intermediates that lie within metabolic pathways. Assuming a standard linear metabolic pathway that involves the stepwise chemical conversion of a series of metabolites, the loss of an enzyme that converts toxic metabolite “b” to a nontoxic metabolite “c” may result in the accumulation of “b” to toxic levels in the cell (Fig. 1c). This approach may overcome some of the aforementioned limitations of the building block deprivation approach. Even if alternate synthesis or uptake routes exist for the building block synthesis, it cannot remedy the issue of accumulated toxic metabolites. Importantly, if this metabolic pathway is more active in a particular type of cancer cell compared with a normal cell, then the accumulation of “b” should occur at a greater rate in the cancer cell, providing a therapeutic window. The more toxic metabolite “b” is, the more likely it is that the disruption of the downstream enzyme will be detrimental to the cancer cell.

First, we describe many metabolites normally produced in our cells that have highly toxic properties. We illustrate potential mechanisms for their toxicity, such as the presence of highly reactive groups that allow them to covalently modify cellular components, or their similarity to other metabolites that make them de facto competitive inhibitors. Second, we describe recent findings that demonstrate this approach, with an emphasis on the selenocysteine biosynthesis pathway, which we have recently shown to allow cancer cell-specific poisoning. Finally, we discuss future directions for applying this approach across many metabolic pathways to uncover novel Achille’s heels in different cancers.

## Toxic metabolites

**Fig. 2:**
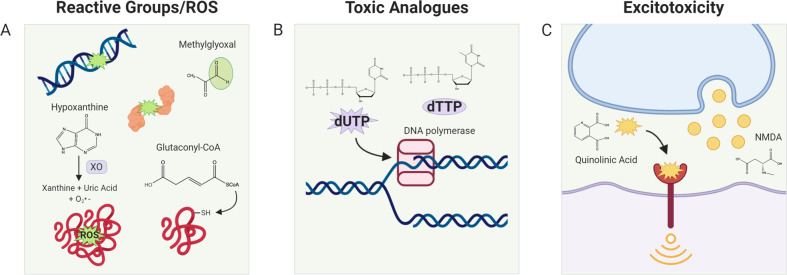
Different categories of toxic metabolites based on their toxicity mechanism. **a** ROS/reactive groups. Some metabolites contain reactive groups such as aldehydes which allow them to form adducts with macromolecules such as DNA and proteins. Others can form reactive oxygen species and similarly damage DNA and proteins through oxidation. The representative ROS forming/reactive metabolites are depicted in the figure. Hypoxanthine is a ROS former, as it produces ROS as it is catabolized. Methylglyoxal is a reactive aldehyde. Glutaconyl-CoA is a reactive metabolite that preferentially reacts with thiol groups. **b** Toxic analogs. Due to the promiscuity of metabolic enzymes and structural similarity of small metabolites, several metabolites confer their toxicity to cells via misincorporation of wrong nucleotides or competitive inhibition of the enzyme reactions. High dUTP/dTTP ratio promotes misincorporation of dUTP, which leads to DNA damage and eventually cell death **c** excitotoxicity. Several metabolites can act as a ligand to neurotransmitters and induce hyperactivation of receptor-mediated signaling, leading to cell death. Chemical structures of metabolites were drawn with ChemDraw software.

It may seem counterintuitive that endogenously produced metabolites, which in many cases form the building blocks of the cell, can be poisonous. However, when cross referencing known metabolites with toxicity databases such as TOXNET [18] or HSDB [19], it becomes clear that many metabolites have toxic profiles. As a striking example, the commonly utilized fixative and hazard substance formaldehyde is produced naturally in the brain via oxidative breakdown of the folate backbone [20]. It can be inferred that under normal physiological conditions, the levels of potentially toxic metabolites in the cell are maintained at nontoxic levels either via further metabolism by metabolic enzymes or efficient secretion mechanisms. Demonstrating this notion, many inborn metabolic disorders such as phenylketonuria or maple syrup urine disease, which involve loss of function mutations in amino acid breakdown genes, are thought to be caused by toxic accumulation of amino acid degradation pathway metabolites, as dietary amino acid restriction can ameliorate or prevent the pathology [21, 22]. Here, we will discuss metabolites that are normally produced in our bodies and are thus endogenous, that also have documented toxic properties. We define “endogenous metabolites” as those that are annotated by Kyoto Encyclopedia of Genes and Genomes [23] as substrates or products of the ~1900 metabolic enzymes encoded in our genome [24]. It is clear in the body of literature that there are documented toxic properties for many of these metabolites. Those defined toxic metabolites could be categorized based on how they confer the toxicity to the cells (Fig. 2); (1) ROS-producing metabolites, (2) reactive metabolites, (3) metabolite analogs, (4) excitotoxins, and (5) not established/unknown biology.

## ROS-producing metabolites

The major intracellular sources of reactive oxygen species (ROS; OH•, O_2_^−•^, H_2_O_2_, etc.) are thought to be mitochondrial respiration and the activities of NADPH oxidase, 2-oxoacid dehydrogenases, and superoxide dismutases (SOD1/2/3) [25]. In a normal state, the intracellular level of ROS is controlled in balance with intracellular antioxidants to protect cells from oxidative damage [25, 26]. It appears that intermediates of metabolic pathways can also directly or indirectly result in ROS production, which may account for their toxicity (Fig. 2a). For example, hydrogen selenide is an intermediate in the selenocysteine biosynthesis pathway, a pathway that incorporates the trace nutrient selenium to provide selenocysteine residues in 25 different selenoproteins in the human body. Inhalation of hydrogen selenide is known to be toxic [27], and it has been proposed that selenide reacts with water during its decay into elemental selenium, forming hydroxy radicals, superoxide and hydrogen peroxide [28], suggesting a potential mechanism for its toxicity. Bilirubin is a metabolite in the heme breakdown pathway, which occurs in the liver and is proposed to be the toxic component of jaundice [29]. While bilirubin can function as an antioxidant at high doses, it has also been shown to result in mitochondrial ROS accumulation [30, 31], which promotes neuronal and cancer cell death [32, 33]. Treatment of a high level of hypoxanthine, a product of the purine catabolism pathway, is also cytotoxic by creating superoxide radicals as it breaks down to xanthine and uric acid by xanthine oxidase [34, 35]. Polyamines in the arginine catabolic pathway such as agmatine [36, 37], spermidine [38], and spermine [39, 40] can directly bind to acidic sites in proteins and nucleic acids and also generate hydrogen peroxide when they are processed via a number of oxidases [41], which may account for their toxicity. 3-hydroxyanthranilate, an intermediate in the tryptophan degradation pathway, is known to kill T cells through ROS generation [42]. Autooxidation of the 3-hydroxyanthranilate requires molecular oxygen, which produces superoxide radicals and hydrogen [43]. In addition, cysteamine, an amino thiol compound and an intermediate of the taurine synthesis pathway, has been shown to inhibit cell proliferation and survival and cancer cell invasion and metastasis at high levels [44, 45]. A proposed mechanism is that its thiol group can produce hydrogen peroxide upon reaction with transition metals [46].

## Reactive metabolites

Some metabolites contain functional groups that are chemically reactive. As such, they can form adducts or react with macromolecules such as DNA and proteins, which can result in their detrimental modification and cellular toxicity (Fig. 2a). The aldehydes are molecules having at least one hydrogen atom substituent on the carbonyl carbon atom. These metabolites are toxic due to their electrophilicity by creating adducts with biological nucleophiles [47]. For example, formaldehyde, formed during the oxidative breakdown of the folate backbone, has the well-known toxic property of being able to efficiently crosslink biological macromolecules [20]. In addition, malondialdehyde and 4-hydroxynonenal (4-HNE), also known as lipid peroxidation markers because they are final products of polyunsaturated fatty acid peroxidation [48], can confer toxicity to cells by interacting with DNA and proteins [49–51]. Other lipid peroxidation products that have aldehyde groups, such as crotonaldehyde, 4-oxononenal (4-ONE), and acrolein have been reported as toxic molecules [51–53]. Both 4-HNE and 4-ONE are potent cross-linkers of tubulin assembly, which results in impairment of tubulin function [51]. Acrolein forms DNA and protein adducts and promotes mitochondrial disruption, ER stress, and membrane damage at the cellular level [53]. The dopamine catabolic pathway metabolite 3,4-dihydroxyphenylacetaldehyde is also a toxic aldehyde, as it modifies tyrosine hydroxylase and inhibits its activity [54]. As another example of aldehydes, methylglyoxal can be produced nonenzymatically as a byproduct of excessive glycolytic intermediates or glycine, and via other mechanisms, and is a potent toxic compound [55–58]. Propensity for methylglyoxal production may be high in cancer cells as its upstream sources of glycolysis and glycine production are upregulated in cancers. Showing another important aspect of methylglyoxal in tumor biology, regulatory myeloid cells suppress T-cell effector function via cell–cell transfer of methylglyoxal, aiding the immune evasion of tumors [59]. Beyond cancer biology, methylglyoxal is implicated as a pathological agent in diabetes and neurodegenerative disorders such as Alzheimer’s and Parkinson’s disease [58].

Several metabolites are toxic due to their strong tendency to react with free thiol groups of proteins or glutathione (Fig. 2a). For example, methacrylyl-CoA, an intermediate of the valine catabolic pathway, is proposed to be a toxic component of beta-hydroxyisobutyryl coenzyme A deacylase deficiency [60]. Methacrylyl-CoA readily reacts with free sulfhydryl groups, which results in cellular damage [60, 61]. Glutaconyl-CoA, another intermediate of the valine catabolic pathway, has been suggested to be the main toxic component of the inborn disorder glutaryl-CoA dehydrogenase deficiency [62]. The addition product of glutaconyl-CoA to cysteine has been found in urine [62]. As another example of a reactive metabolite, xanthurenic acid, an end product of the tryptophan degradation pathway, is also toxic by covalently binding to proteins, which leads to protein unfolding, ER stress [63], and apoptosis [64].

Overall, many examples exist in the literature of metabolites that show reactivity toward biological macromolecules and thus exert their toxicity. In addition to further exploring the examples mentioned above, it may be possible in future efforts to predict toxic metabolites based on the presence of reactive groups, such as reactive aldehydes, and to explore these as a therapeutic strategy.

## Toxic analogs

Many enzymes have the capacity to catalyze not just one standard reaction, but also physiologically irrelevant secondary reactions, referred to as “promiscuous” reactions [65, 66]. These promiscuous reactions of enzymes have provided an opportunity to develop “antimetabolite” chemotherapeutic drugs such as nucleoside analogs [67] and glucose analogs [68]. These analogs compete with physiological glucose and nucleosides and bind to intracellular enzymes to induce cytotoxicity via behaving like antimetabolites. Interestingly, some findings support that the accumulation of endogenous metabolites can induce cell death by working as antimetabolites. For example, elevated dUTP/dTTP ratios can cause uracil misincorporation by DNA polymerase during DNA synthesis [69, 70] (Fig. 2b). The misincorporation of dUTP leads to DNA excision and strand breakage, which eventually leads to cell death [71]. Another example of a potential toxic metabolite analog is S-adenosylhomocysteine (SAH). SAH is a product of a methyltransferase reaction that uses S-adenosylmethionine (SAM) as a substrate, and it is structurally analogous to SAM. It is well established that the ratio of SAM/SAH is an indicator of cellular methylation potential and SAH accumulation can inhibit the activity of many methyltransferases via feedback inhibition [72–75]. Supporting that the disrupted methylation status could be detrimental to cells, the accumulation of SAH was shown to be toxic to lymphoma and lymphoblastic cells [73, 74]. 2-Hydroxyglutarate (2-HG) is another example of a toxic analog metabolite, as it is chemically similar and acts as an analog to alpha-ketoglutarate (α-KG). 2-HG exists in D and L enantiomeric forms. D-2-HG is produced by neomorphic mutations in isocitrate dehydrogenase (IDH) 1 or 2 which causes them to aberrantly produce D-2-HG from α-KG. D-2-HG in turn is an oncometabolite that is responsible for the oncogenic effects of IDH mutations. L-2-HG is formed via promiscuous, NADH-dependent substrate usage of lactate dehydrogenase A and malate dehydrogenase 1/2, due to increased NADH/NAD+ ratio, which occurs in hypoxia or electron transport chain dysfunction [76–78]. L-2-HG can also accumulate by defects in the L-2-HG processing enzyme L-2-hydroxyglutarate dehydrogenase (L2HGDH) and is the cause of the disorder 2-hydroxyglutaric aciduria [79]. Both of these forms can potentially be both oncogenic as well as toxic due to the inhibition of α-KG-dependent histone and DNA demethylases and resulting changes in gene expression [80]. Indeed, the accumulation of L-2-HG, due to L2HGDH mutations or mitochondrial stress, exerts neuronal or glial dysfunction and toxicity in the central nervous system [81–83]. The prevention of L-2-HG overproduction through electron transport chain activity is also important for the normal function of hematopoietic stem cells and regulatory T cells [77, 78]. It is intriguing to consider that some toxic metabolites that are endogenously produced in cancer cells may be used to poison them via a mechanism similar to the aforementioned examples of “antimetabolite” chemotherapeutics.

## Excitotoxins

Neurotransmitters are endogenous chemicals that transmit signals from one neuron to another neuron, gland cell, or muscle cell [84]. The identified neurotransmitters are mostly amino acids, peptides, and monoamines, and their binding to the receptor can activate calcium influx and/or downstream signaling. Some endogenous metabolites have the potential to target neurotransmitter receptors, and they can excessively activate this process to toxic levels. Some toxic metabolites appear to exert toxicity by aberrantly triggering neurotransmitter receptors. For example, quinolinate, a downstream product of the kynurenine processing pathway, is a neuroactive metabolite. Quinolinic acid induces excitotoxic cell damage as an agonist for the N-methyl-D-aspartate (NMDA) receptor (Fig. 2c), and additional mechanisms such as oxidative stress, cytoskeletal disruption, energetic dysfunction may also be involved [85, 86]. Glutaryl-CoA dehydrogenase deficiency, an inherited neurometabolic disorder, is characterized by the accumulation of glutaric acid and 3-hydroxyglutaric acid [87, 88]. The 3-hydroxyglutaric acid induces cell death via NMDA receptor activation, and both glutaric acid and 3-hydroxyglutaric acid regulate glutamatergic and GABAergic neurotransmission, which leads to disrupted balance of excitatory and inhibitory neurotransmission [88]. As another example of a possible excitotoxin, homocysteine activates NMDA receptor-mediated signaling [89, 90]. Homocysteine also acts as a reactive metabolite by incorporating into protein via disulfide or homocysteinylation on amide groups, which affects protein structure and function [91]. While these toxic metabolites may exert toxicity in a neuronal context, some studies suggest that excitotoxic mechanisms can also affect cancer cells. It has been recognized that many types of cancer cells ectopically express neurotransmitters [92], and some studies indicate toxic effects of neurotransmitters/excitotoxic metabolites such as dopamine and the aforementioned 3-hydroxyglutaric acid [93]. Undesirable toxicity to neurons is an obvious concern for strategies which aim to poison cancer cells with these types of toxic metabolites. It may nonetheless be feasible to explore such a therapeutic strategy for inducing accumulation of these metabolites in tumors outside the CNS, in which the drug cannot cross the blood brain barrier, thus avoiding CNS toxicity.

## Not established (unknown biology)

Some metabolites have been known to have antiproliferative or toxic effects in cells, but their toxic mechanisms are not clearly understood. For example, dehydroepiandrosterone, an intermediate for steroid hormone synthesis, exerts growth inhibitory functions in hepatocytes [94], neuronal cells [95, 96], and breast and pancreatic cancer cells [97, 98]. The growth inhibitory effect of the dehydroepiandrosterone appears to involve perturbation of complex I of the mitochondrial respiratory chain [95], DNA synthesis [94], and SAM production and cardiolipin depletion [97], However, how these processes are inhibited are not clear. Ceramide, a central metabolite in sphingolipid metabolism, is well established as a tumor suppressor lipid and a cell death-inducing signal, and perhaps the best known “toxic metabolite” [99]. Ceramide has been shown to affect various processes including ERK and JNK signaling and modulation of apoptotic machinery [100], but the exact mechanism for how ceramide impacts these biological processes is still under debate. Accumulation of squalene, an intermediate in sterol biosynthesis, has been shown to be lipotoxic in yeast and cancer cells [101–103] by disturbing the maintenance of plasma membrane potential and increasing passive uptake [103], but further molecular characterizations are required. As another example, 4-methylthio-2-oxobutanoate, an intermediate in the methionine salvage pathway, has been shown to effectively induce apoptosis, and the underlying mechanism for this effect is unclear [104, 105]. Fumarylacetoacetate, a metabolite in the tyrosine degradation pathway that is thought to be the pathogenic metabolite in hereditary tyrosinemia [106, 107], has been shown to be both mutagenic and cytotoxic, eliciting deregulation of various biological processes such as glutathione depletion, ERK activation, and cytochrome c release [108, 109]. While some mechanisms such as reactivity with thiols and mitotic spindle disruption have been suggested [109], how fumarylacetoacetate interacts with these biological processes is not established. Another set of metabolites for which the mechanism of toxicity is incompletely understood are the gangliosides, sialic acid containing glycosphingolipids, which are documented to be toxic and are associated with inherited diseases due to its toxicity [110, 111]. While aberrant lysosomal accumulation and dysfunction, signaling dysfunction, and mechanisms related to lipid raft-like microdomains have been uncovered, the exact molecular nature of ganglioside toxicity remains to be found. While we are showing some selected examples, it appears that there are many reports of toxic effects of metabolites spanning various metabolic pathways, for which the reason for toxicity is undetermined. The fact that these metabolites can be toxic when at aberrantly high levels may indicate that they normally have previously unrecognized functions as regulatory components of biological processes or similarly interact with proteins or other biomolecules. An intriguing possibility is that, such as in ceramide’s ability to act as a potent death signal, there may exist important signaling functions for many metabolites that are yet to be uncovered.

As seen across the body of literature and summarized above, a variety of metabolites can be toxic to cells through various molecular mechanisms known and unknown. There are likely many other metabolites and mechanisms of toxicity yet to be uncovered. These examples lead us to posit that many metabolic enzymes in cells may be important not necessarily for what they produce, but for what they get rid of.

## The kitchen sink and achieving cancer-specific toxicity

As outlined in the previous section, many metabolites that are normally produced in cells have toxic properties, suggesting that their accumulation would be deleterious to a cell. This has an important implication for the function of metabolic enzymes—that enzymes that process a toxic metabolite in a pathway may serve an important detoxification function, as they prevent accumulation of toxic metabolites. This immediately suggests that it may be possible to induce poisoning of cancer cells with an accumulation of toxic metabolites that they themselves have produced, by targeting such “detoxification enzymes”. Thus, the detoxifying enzymes represent novel and druggable targets for killing cancer cells.

**Fig. 3:**
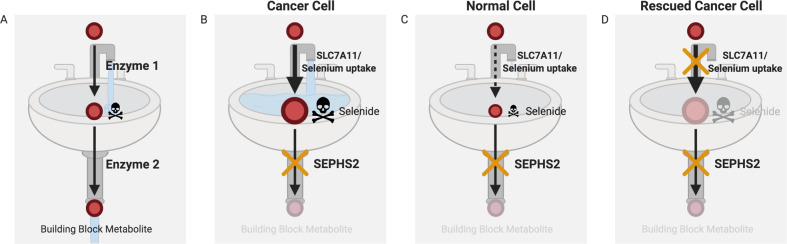
Kitchen sink model for metabolic pathway containing toxic metabolite. Red dots and arrows represent metabolites and enzymatic reactions, respectively. Faucet and drain of kitchen sink are analogies of enzymatic reactions of upstream and downstream metabolic enzymes, respectively; the basin represents the accumulation level of the toxic metabolite. **a** The level of toxic metabolite candidate is constantly maintained at a nontoxic level as both faucet and drain are opened (active), balancing metabolite production and removal. **b** Example of toxic metabolite accumulation by targeting the downstream detoxification enzyme. SLC7A11 and SEPHS2 are the faucet and drain for toxic hydrogen selenide in the selenocysteine biosynthesis pathway. Many cancer cells have elevated SLC7A11 expression relative to normal cells and are able to import selenium and produce selenide at an elevated rate. Thus, their faucet is wide open, and disrupting SEPHS2 is akin to blocking the drain, resulting in toxic overflow. **c** However, in normal cells, disrupting SEPHS2/blocking the drain is not a problem as the faucet is relatively “closed”. In both **b** and **c**, building block metabolite cannot be produced, yet only **b** is toxic, indicating that the toxicity must have come from toxic metabolite accumulation rather than consequences of downstream building block production. **d** Similarly, in a cancer cell such as in scenario **b**, the preemptive KO of the upstream transporter SLC7A11 rescues the cells against the toxic effects of SEPHS2 KO, further demonstrating that toxicity in **b** was due to toxic metabolite accumulation. All diagrams were created with BioRender.com.

But how can a therapeutic window exist for such a mechanism, so that the toxicity occurs primarily in cancer cells? In theory, the detoxification requirement would be in proportion to the production of the toxic metabolite in the first place. This can be analogized as a “kitchen sink” model (Fig. 3), where the faucet represents the upstream metabolic enzymes or transporters that are responsible for formation or import of the toxic metabolite, and the drain represents the “detoxifying” enzyme preventing the overflow of the sink with the toxic metabolite. In this model, the drain is only required in cells in which the faucet is on, i.e., the toxic metabolite is appreciably being produced. Thus, if certain cancer cells produce a particular toxic metabolite at a greater rate than normal cells (Fig. 3b, c), they should be more dependent on the detoxifying enzyme than normal cells, and drugs that target this detoxifying enzyme should be selectively toxic toward these cells.

Our recent findings on the selenocysteine biosynthesis pathway [112] illustrate and provide a proof of principle of the kitchen sink concept of selective toxicity. This metabolic pathway synthesizes selenocysteinyl-tRNA used in the production of 25 different selenoproteins encoded in the human genome, which contain one or more selenocysteine residues. A metabolic intermediate in this pathway, hydrogen selenide, is reported to be toxic [27, 113] and in the class of ROS-producing toxic metabolites (Table 1). We found that the sole enzyme that processes selenide—SEPHS2—is essential in a subset of cancer cell lines, including breast cancer and glioblastoma lines, but not in any nontransformed or primary lines tested. Investigating further, we were able to conclude that SEPHS2 is essential in these cell lines due to their elevated expression of SLC7A11, which induces the import of the dietary selenium compound selenite and its conversion to toxic selenide (Fig. 3b). Normal cells are spared, as they do not appreciably express SLC7A11, and thus do not depend on SEPHS2 detoxification (Fig. 3c). Importantly, we verified a toxic gain-of-function mechanism of toxicity based on selenide toxicity, as preemptive KO of SLC7A11, while also abrogating selenoprotein production similarly to SEPHS2 KO, prevented the toxicity of SEPHS2 KO in cancer cells. This exemplifies a critical and robust test for identification of a toxic metabolite poisoning event: disruption of the upstream toxic metabolite producing step should rescue against the effects of detoxifying enzyme KO, if the detoxifying enzyme was required for detoxification rather than what it produces (Fig. 3d).

**Table 1:** List of toxic metabolites.

Category	Toxic metabolite	Related pathway	Ref supporting toxic property
ROSgens	Hydrogen selenide	Selenocysteine synthesis	[27, 28, 113]
	Bilirubin	Heme degradation	[29–33]
	Hypoxanthine	Purine metabolism	[34, 35]
	Agmatine	Arginine catabolism	[36, 37]
	Spermidine	Arginine catabolism	[38, 41]
	Spermine	Arginine catabolism	[39–41]
	3-Hydroxyanthranilate	Tryptophan degradation	[42, 43]
	Cysteamine	Taurine synthesis	[44–46]
Reactive metabolites (Aldehydes, reactive to thiols, etc.)	Formaldehyde	By product of oxidative demethylation/deamination	[20, 115]
	Malondialdehyde	Lipid peroxidation byproduct	[49]
	4-hydroxynonenal	Lipid peroxidation byproduct	[50, 51]
	Crotonaldehyde	Lipid peroxidation byproduct	[52]
	4-oxononenal	Lipid peroxidation byproduct	[51]
	Acrolein	Lipid peroxidation byproduct	[53]
	3,4-dihydroxyphenylacetaldehyde	Dopamine degradation	[54]
	Methylglyoxal	Various	[55–59]
	Methacrylyl-CoA	Valine catabolism	[60, 61]
	Glutaconyl-CoA	Valine catabolism	[62]
	Xanthurenic Acid	Tryptophan degradation	[63, 64]
Toxic analogs	dUTP	Nucleotide synthesis	[69–71]
	S-adenosylhomocysteine	Transmethylation	[72–74]
	L-2-hydroxyglutarate	Promiscuous reaction of LDH and MDH	[77–83]
Excitotoxins	Quinolinic acid	Kynurenine degradation	[85, 86]
	3-hydroxyglutaric acid	Kynurenine degradation	[87, 88]
	Glutaric acid	Kynurenine degradation	[88]
	Homocysteine	Homocysteine synthesis	[89–91]
Not Established	Dehydroepiandrosterone	Steroid hormone synthesis	[94–98]
	Ceramide	Sphingolipid synthesis	[99, 100]
	Squalene	Steroid hormone synthesis	[101–103, 116]
	4-methylthio-2-oxobutanoate	Methionine salvage pathway	[104, 105]
	Fumarylacetoacetate	Tyrosine degradation	[107–109]
	Gangliosides	Ganglioside synthesis	[110, 111]
	ex) Ganglioside GM3	Ganglioside synthesis	[117–119]
	ex) Ganglioside GD3	Ganglioside synthesis	[120–122]

Thus, the SLC7A11/selenide production/SEPHS2 detoxification axis illustrates a kitchen sink mechanism of toxicity, and at the same time suggests potential advantages of a metabolite poisoning strategy to kill cancer cells. First, this pathway illustrates how in some instances the downstream building block product of a metabolic pathway per se may not be critical for the survival and proliferation of a cell, in contrast to the acute requirement for detoxification. Indeed, we demonstrated that the loss of all selenoproteins can be tolerated in normal cells indefinitely, whereas the toxic accumulation of selenide can acutely induce cell death in cancer cells. Second, it demonstrates how the factor that determines the requirement for detoxification is likely to be logical and predictable—in this case, the transporter involved in both selenium import and selenide production. This is a significant advantage, as one can easily predict which types of cancers may be amenable to SEPHS2 inhibitor treatment, either at the level of the cancer subtype or even at the individual patient level. This will help predict exactly which patients will benefit most from pharmacological inhibition of the detoxifying enzyme. Third, the level of toxic metabolite accumulation may be modulated by dietary input into the pathway, which can help increase or decrease efficacy to “dial in” the correct dosage of the toxic metabolite. We showed that supplementation with selenite in the tissue culture media synergistically enhanced the toxicity of SEPHS2 KO in cancer cells, suggesting that with this type of approach, pharmacological inhibition of the detoxifying enzyme may be combined with dietary supplementation with metabolic precursors to the toxic metabolite. Conversely, if the toxic metabolite accumulation from cancer cells is too great as to pose hazards to the patient, the toxicity may be tempered through dietary restriction of the upstream precursors. In conclusion, the toxic metabolite accumulation approach provides a method to induce acute poisoning to kill cancer cells, with additional potential advantages of predictability of susceptible cells and the opportunity for dietary modulation of the treatment.

## Discussions: caveats and a future blueprint for expansion

We have discussed in “The kitchen sink and achieving cancer-specific toxicity” the concept, proof of principle, and advantages of a toxic metabolite accumulation strategy to kill cancer cells. As a novel concept, this approach holds promise for uncovering novel classes of cancer targets and even clinical implications for diseases beyond cancer; however, there are also important caveats to consider. Here, we will discuss how this approach can be expanded in a systematic manner, but with caution.

As described in “Toxic metabolites”, examples of toxic metabolites can be found sprinkled throughout the literature across various fields. In each case, the downstream enzyme(s) that are responsible for processing these metabolites are “detoxifiers” and potential targets for inducing toxicity in cells, and this may be selective toward certain cancer cells if they have an increased production rate for the toxic metabolite. Thus, it will be useful to catalog an extensive list of toxic metabolites (the “endotoxome”) and the set of downstream enzymes that process them. In this manner, once an extensive list has been curated, it may be possible to systematically analyze the metabolic activities of different cancers and use this to predict which toxic metabolite pathways can be targeted to achieve selective toxicity for a particular cancer. This sort of analysis may even be carried out on a patient to patient or individual tumor to tumor level based on gene expression analyses or metabolite profiling analyses of tumor biopsies. We predict that in the coming years many more studies and strategies involving individual detoxification enzymes may be uncovered, and over time systematic approaches may be developed.

An important caveat to a systematic identification of toxic metabolites is that it is hard to determine an exact threshold to define a toxic metabolite vs a nontoxic metabolite. Even relatively nonreactive, nontoxic metabolites may become toxic at high enough concentrations. For systematic cataloging of toxic metabolites, empirical dose response testing of each “endogenous metabolite” in purified form across several types of cell lines would allow one to set a dose-defined threshold or hierarchy of toxic metabolites. However, procuring an extensive library of metabolites in purified form presents a significant challenge to this approach. As an alternative approach, toxic metabolites may be “functionally” defined and cataloged using the “kitchen sink” model. As described in “The kitchen sink and achieving cancer-specific toxicity”, if KO of a metabolic enzyme is deleterious, but can be rescued by the concomitant KO of the upstream enzyme (or transporter), this would indicate that the accumulation of the metabolite between those two enzymes was the cause for the toxicity (Fig. 3d). While functionally determining these cases represents a challenge in terms of experimental scale, mining the essentiality of enzymes in large-scale essentiality data resources such as the Cancer Cell Line Encyclopedia [114] may be a useful starting point.

A second caveat to be considered in future studies is the transport of toxic metabolites into and out of cells. Certain toxic metabolites may be incredibly toxic inside cells but may not be easily identified, as media treatment of the metabolite may not readily enter cells to exert a toxic effect. The same metabolites that are not easily transported into and out of a cell may represent attractive targets for therapy as they are “trapped” within the cell, as opposed to another metabolite that may be readily exported from the cell before toxic accumulation is achieved.

The third caveat is possible host toxicity. Even though the accumulation of a toxic metabolite should occur in a cancer cell-specific manner as described, it is possible that the toxic metabolites that accumulate in the tumor may spread to the host tissues. This risk further emphasizes the need to test examples of this approach in vivo, for example in tumor xenograft models. Conversely, the “spread” of toxicity may actually be a significant advantage in therapy when considering that tumors are known to be metabolically heterogeneous; thus, if a majority of tumor cells are selectively vulnerable to toxic metabolite accumulation, this may result in toxicity of neighboring invulnerable tumor cells, overcoming a universal limitation of chemotherapy.

Despite these potential caveats, the notion of poisoning cancer cells with their own metabolites represents an unconventional, underexplored, and potentially highly efficacious method for killing cancer cells in a predictive and selective manner. We hope that this review serves as a blueprint for other groups to determine whether toxic metabolite exploitation opportunities exist in their metabolic pathway of interest. Furthermore, the relevance of toxic metabolites may extend beyond simply being an exploitable tool in cancer therapy. Since toxic metabolites are normally produced, their presence—however transient—may contribute to cellular wear and tear mechanisms. The formation of aldehydes and ROS-producing metabolites, for example, if reacting with DNA, may contribute to the daily burden of DNA damage in a cell. If they react with proteins, this can result in protein dysfunction and even misfolding events. DNA damage and protein misfolding are events thought to be universally present (and pathologically contributing) to aging and age-dependent disorders such as Alzheimer’s disease. Any deregulation of transcription, transport, or other biological functions that result in an imbalance between toxic metabolite production and detoxification may therefore increase the level of cellular damage over time in an organism. Toxic metabolites may represent a large and underappreciated group of metabolites that, over time, can contribute to a variety of disorders; thus, the premises mentioned here may be applicable across a range of diseases. In closing, toxic metabolites may lie at the interface of metabolism and disease in many regards, and as outlined here, cancer is a prime avenue in which they may be harnessed for therapeutic benefit.
